# Regulation of DNA synthesis and the cell cycle in human prostate cancer cells and lymphocytes by ovine uterine serpin

**DOI:** 10.1186/1471-2121-9-5

**Published:** 2008-01-24

**Authors:** Maria B Padua, Peter J Hansen

**Affiliations:** 1 Department of Animal Sciences, University of Florida, Gainesville, FL 32611-0910, USA

## Abstract

**Background:**

Uterine serpins are members of the serine proteinase inhibitor superfamily. Like some other serpins, these proteins do not appear to be functional proteinase inhibitors. The most studied member of the group, ovine uterine serpin (OvUS), inhibits proliferation of several cell types including activated lymphocytes, bovine preimplantation embryos, and cell lines for lymphoma, canine primary osteosarcoma and human prostate cancer (PC-3) cells. The goal for the present study was to evaluate the mechanism by which OvUS inhibits cell proliferation. In particular, it was tested whether inhibition of DNA synthesis in PC-3 cells involves cytotoxic actions of OvUS or the induction of apoptosis. The effect of OvUS in the production of the autocrine and angiogenic cytokine interleukin (IL)-8 by PC-3 cells was also determined. Finally, it was tested whether OvUS blocks specific steps in the cell cycle using both PC-3 cells and lymphocytes.

**Results:**

Recombinant OvUS blocked proliferation of PC-3 cells at concentrations as low as 8 μg/ml as determined by measurements of [^3^H]thymidine incorporation or ATP content per well. Treatment of PC-3 cells with OvUS did not cause cytotoxicity or apoptosis or alter interleukin-8 secretion into medium. Results from flow cytometry experiments showed that OvUS blocked the entry of PC-3 cells into S phase and the exit from G_2_/M phase. In addition, OvUS blocked entry of lymphocytes into S phase following activation of proliferation with phytohemagglutinin.

**Conclusion:**

Results indicate that OvUS acts to block cell proliferation through disruption of the cell cycle dynamics rather than induction of cytotoxicity or apoptosis. The finding that OvUS can regulate cell proliferation makes this one of only a few serpins that function to inhibit cell growth.

## Background

Serine proteinase inhibitors (serpins) inactivate their target proteinases through a suicide substrate-like inhibitory mechanism. The proteinase binds covalently to the reactive center loop (RCL) of the serpin and cleaves the scissile bond at the P1-P1' site. The RCL then moves to the opposite side to form the β-sheet A and a distortion in the structure of the proteinase that results in its inactivation [1-3]. Not all serpins, however, exert proteinase inhibitory activity. Some examples are corticosteroid and thyroxine binding globulins, which function as hormone transport proteins [4], the chaperone heat shock protein 47 [5], mammary serine protease inhibitor (Maspin), which increases the sensitivity of cancer cells to undergo apoptosis [6], and pigment epithelium derived factor (PEDF), which has neurotrophic, neuroprotective, antiangiogenic, and proapoptotic actions [7].

Another class of serpins without apparent proteinase activity is the uterine serpins. These proteins, which are produced by the endometrial epithelium of the pregnant cow, sow, sheep, and goat [8-13], have been classified as either a separate clade of the serpin superfamily [14] or as a highly-diverge group of the α1-antitrypsin clade [1]. The best characterized protein of this unique group of serpins is ovine uterine serpin (OvUS). This basic glycoprotein is a weak inhibitor of aspartic proteinases (pepsin A and C) [12,15], but it does not inhibit a broad range of serine proteinases [9,16]. Additionally, amino acids in the hinge region of inhibitory serpins are not conserved in uterine serpins and OvUS behaves different in the presence of guanidine HCl than for inhibitory serpins [13,15].

The biological function of OvUS during pregnancy may be to inhibit immune cell proliferation during pregnancy and provide protection for the allogeneically-distinct conceptus [17]. Ovine US decreases proliferation of lymphocytes stimulated with concanavalin A, phytohemagglutinin (PHA), *Candida albicans*, and the mixed lymphocyte reaction [18-22]. In addition, OvUS decreases natural killer cell cytotoxic activity, abortion induced by poly(I)poly(C) in mice [23] and the production of antibody in sheep immunized with ovalbumin [21]. The antiproliferative actions of OvUS are not limited to lymphocytes. Ovine US decreases development of the bovine embryos and proliferation of mouse lymphoma, canine primary osteogenic sarcoma and human prostate cancer cell lines [24,25].

The mechanism by which OvUS inhibits proliferation of cells is unknown. The protein could block activation of cell proliferation, inhibit the cell cycle at other points or induce apoptosis or other forms of cell death. For the PC-3 prostate cancer line, inhibition of cell proliferation by OvUS might involve reduction in interleukin-8 (IL-8) secretion because of the importance of autosecretion of this cytokine for cell androgen-independent proliferation [26]. The goal of the present study was to evaluate the mechanism by which OvUS inhibits cell proliferation. Using PC-3 cells as a model system, it was tested whether inhibition of DNA synthesis involves cytotoxic action of OvUS, induction of apoptosis or disruption of the IL-8 autocrine loop. It was also tested whether OvUS blocks specific steps in the cell cycle for PC-3 cells and lymphocytes.

## Results and Discussion

### Proliferation of PC-3 cells

**Figure 1:**
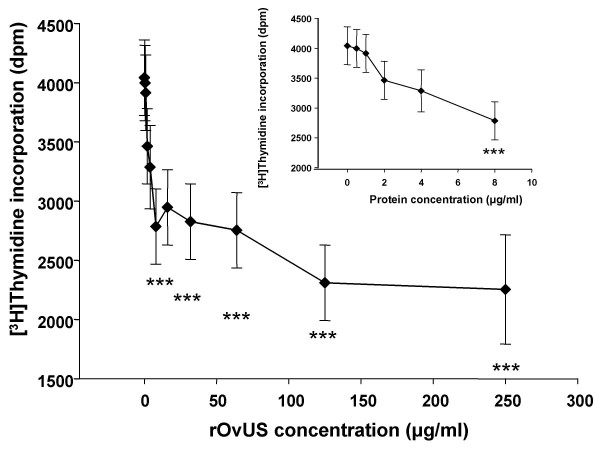
Inhibition of [^3^H]thymidine incorporation of PC-3 cells by recombinant ovine uterine serpin (rOvUS). The inset graph is provided to clarify the effects of rOvUS at lower concentrations (≤ 8 μg/ml). Data represent least-squares means ± SEM. Values that differ from untreated cells are indicated by asterisks (***P < 0.001).

**Figure 2:**
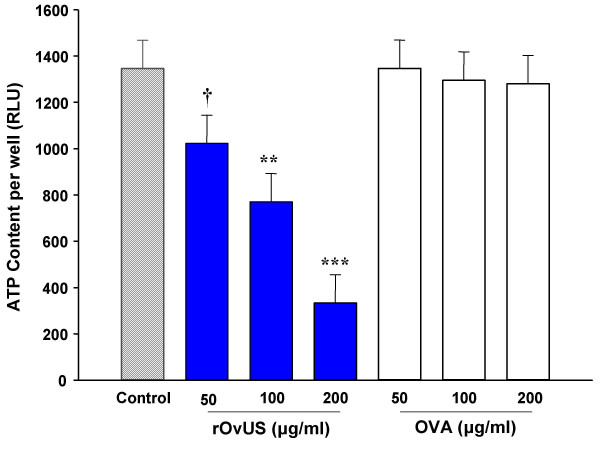
Inhibition of proliferation of PC-3 cells by recombinant ovine uterine serpin (rOvUS) as determined by ATP content/well. Ovalbumin (OVA) was used as a negative control. Data represent least-squares means ± SEM. Means that differ from untreated cells are indicated by symbols (†P < 0.1; **P < 0.01; ***P < 0.001).

The antiproliferative effects of rOvUS on proliferation of PC-3 cells were evaluated by two different assays. In the first experiment, it was shown that rOvUS caused a concentration-dependent decrease in incorporation of [^3^H]thymidine into DNA (P < 0.001) with the minimum effective concentration being 8 μg/ml (Figure 1). The antiproliferative actions of OvUS using [^3^H]thymidine uptake as the measure of proliferation has been demonstrated previously for PC-3 cells and other cell types [18-22,24,25]. To confirm this effect of rOvUS reflected an inhibition in cell proliferation and not a disruption in [^3^H]thymidine uptake by the cells, antiproliferative effects were also evaluated by an assay in which the relative total number of cells per well was estimated by the ATP content per well. Treatment with rOvUS reduced ATP content per well at all concentrations tested (50, 100 and 200 μg/ml) (Figure 2). In contrast, the control serpin, ovalbumin, did not cause effect in the ATP content per well. The finding that rOvUS reduced ATP content per well confirms that the effects of OvUS to reduce [^3^H]thymidine incorporation reflect a reduction in cell proliferation rather than interference with [^3^H]thymidine transport into the cell.

### Lactate dehydrogenase release

**Figure 3:**
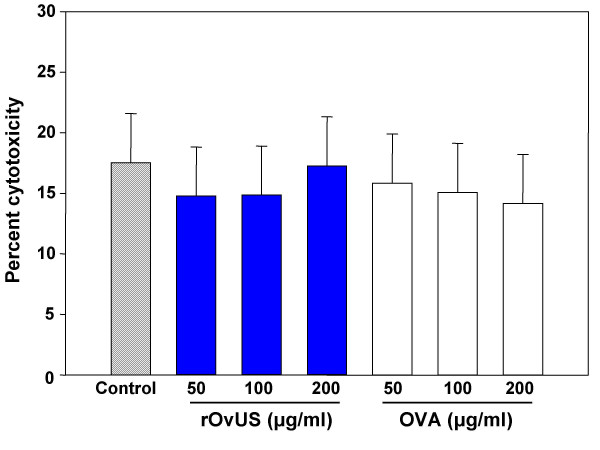
Lack of cytotoxic effect of recombinant ovine uterine serpin (rOvUS) on PC-3 cells was measured by the release of lactate dehydrogenase. Ovalbumin (OVA) was used as a control protein. Data represent least-squares means ± SEM.

Possible cytotoxic effects of rOvUS on PC-3 cells were evaluated by measurements of lactate dehydrogenase release into culture medium (Figure 3). None of the concentrations of rOvUS or OVA tested caused an increase in the percent of lysed cells during culture. Thus, rOvUS does not inhibit proliferation through induction of cell death.

### DNA fragmentation (apoptosis)

**Figure 4:**
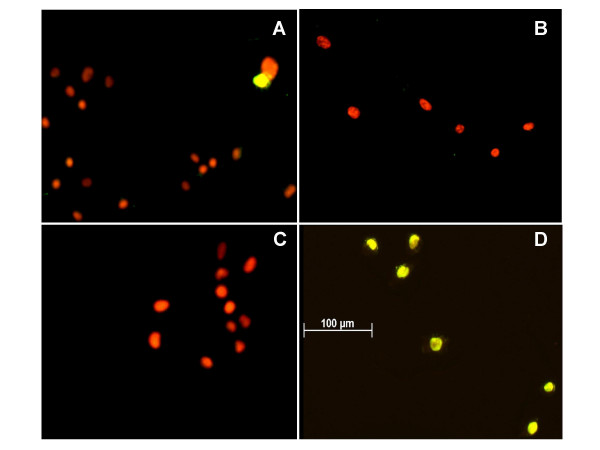
Representative photomicrographs of PC-3 cells labeled using the TUNEL procedure after 48 h of culture with either 100 (A) or 200 μg/ml (B) of rOvUS or 200 μg/ml of the control protein ovalbumin (C). Cells in panel D were treated with DNAse as a positive control.

**Figure 5:**
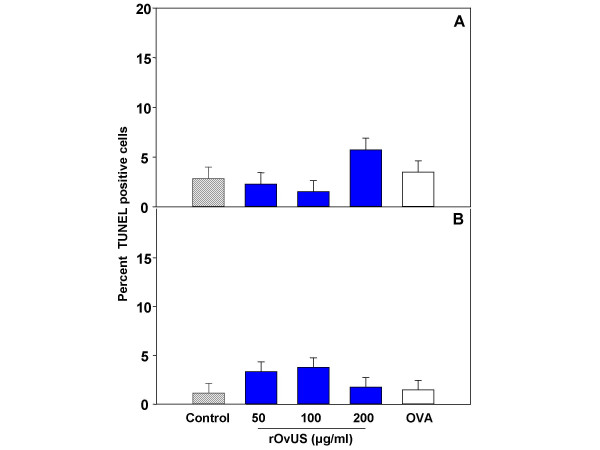
Effect of recombinant ovine uterine serpin (rOvUS) on DNA fragmentation (apoptosis) of PC-3 cells. Results show the percent of TUNEL positive cells at 24 (A) and 48 (B) h after addition of the treatments. Data represent least-squares means ± SEM. Ovalbumin (OVA, 200 μg/ml) was used as control protein.

The TUNEL procedure was used to test whether rOvUS decreased cell proliferation by induction of DNA fragmentation characteristic of apoptosis and other forms of cell death. Representative images of TUNEL labeled cells are shown in Figure 4 and the average percent of cells that were TUNEL positive is shown in Figure 5. Treatment of PC-3 with either rOvUS or the control protein OVA did not increase the percent of cells that were TUNEL positive at either 24 or 48 h after treatment; the percentage of cells that were TUNEL positive was low for all groups (< 5.7%). The fact that rOvUS did not induce apoptosis makes the action of this serpin distinct from that of two other serpins that inhibit cell proliferation. Both maspin [6] and PEDF [7] are proapoptotic serpins.

### Interleukin-8 secretion

**Figure 6:**
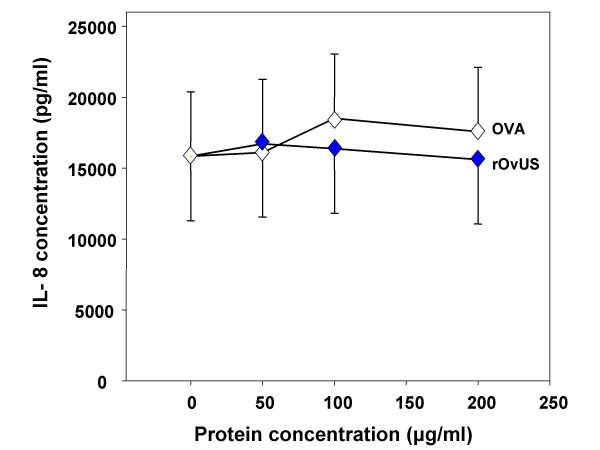
Effect of recombinant ovine uterine serpin (rOvUS) on interleukin (IL) – 8 concentration in cell culture supernatants of PC-3 cells. Ovalbumin (OVA) was used as control serpin. Data represent least-squares means ± SEM.

Interleukin-8 accumulation in the medium was measured because of the autocrine effect of this chemokine on prostate cell proliferation [26]. In addition, at least one class of molecule that inhibits PC-3 cell proliferation, soy isoflavones, also reduces IL-8 secretion [27]. As shown in Figure 6, there was, however, no effect of rOvUS on accumulation of IL-8 into conditioned cultured medium. Thus, rOvUS does not block PC-3 cell proliferation through inhibition of IL-8 secretion.

### Cell cycle dynamics

**Figure 7:**
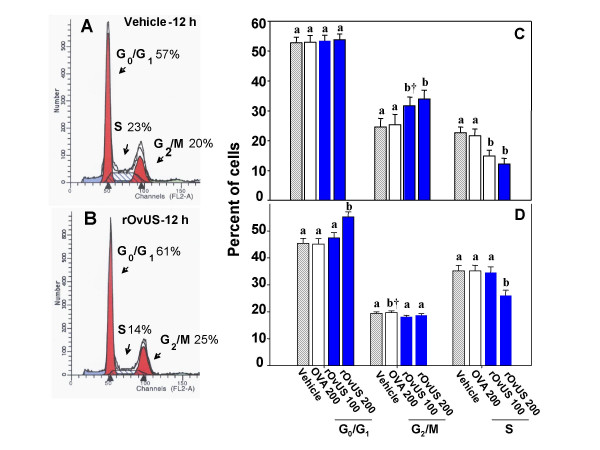
Cell cycle dynamics of PC-3 cells as affected by recombinant ovine uterine serpin (rOvUS). Controls included vehicle (control) and ovalbumin (OVA). Representative DNA histograms for analysis at 12 h after treatment with vehicle or 200 μg/ml rOvUS are shown in panels A and B, respectively. The least-squares means for results of three separate assays are shown in panels C and D for analysis at 12 h (C) and 24 h (D) after treatment. Bars with different superscripts differ († P < 0.10, others at P < 0.05 or less).

Dynamics through the different phases of the cell cycle were affected by the treatment of PC-3 cells with rOvUS. Representative DNA histograms after treatment with vehicle or 200 μg/ml rOvUS are shown in Figures 7A and 7B while least-squares means ± SEM for for results at 12 and 24 h after treatment are shown in Figures 7C and 7D, respectively. At 12 h after addition of treatments, rOvUS decreased (P < 0.1 and P < 0.05 for 100 and 200 μg/ml of rOvUS respectively) the percent of cells in S phase and increased (P < 0.01) the percent of cells in the G_2_/M phase (Figure 6C). There was no effect of rOvUS on the percent of cells in G_0_/G_1_. At 24 h after addition of treatment, 200 μg/ml rOvUS increased the percent of cells in G_0_/G_1 _(P < 0.001), decreased the percent of cells in S phase (P < 0.01), and did not affect the percent of cells in G_2_/M phase (Figure 6D).

**Figure 8:**
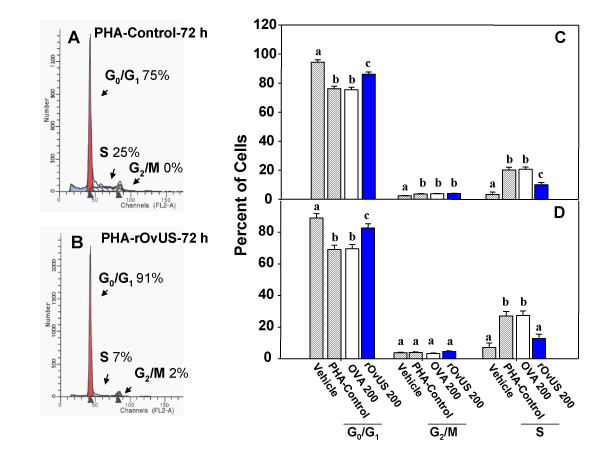
Cell cycle dynamics of lymphocytes as affected by recombinant ovine uterine serpin (rOvUS). Controls included vehicle (control), phytohemagglutinin (PHA) and ovalbumin (OVA). Representative DNA histograms for analysis at 72 h after treatment with PHA or 200 μg/ml rOvUS are shown in panels A and B, respectively. The least-squares means for results of four separate assays are shown in panels C and D for analysis at 72 h (C) and 96 h (D) after treatment. Bars with different superscripts differ (P < 0.05 or less).

Control of the cell cycle dynamics by rOvUS was also evaluated in a second cell type – the peripheral blood lymphocyte. Representative DNA histograms for PHA-treated lymphocytes are shown for control cells and cells treated with 200 μg/ml rOvUS in Figures 8A and 8B, respectively while least-squares means ± SEM are shown in Figures 8C and 8D. At both 72 (Figure 8C) and 96 h (Figure 8D) after addition of PHA, rOvUS increased (P < 0.001) the proportion of lymphocytes in the G_0_/G_1 _phase and decreased (P < 0.05) the proportion of cells in the S phase. In contrast, there was no effect of the control protein (OVA) on the distribution of cells in the cell cycle.

These results indicate that OvUS block cell proliferation through cell cycle arrest in both PC-3 cells and lymphocytes. The differences in specific stages at which the cell cycle was blocked between PC-3 cells and lymphocytes is likely to be caused by differences in activation and regulation pathways for these two cell types. Unlike PC-3 cells, lymphocytes are arrested at G_0 _until proliferation is induced by addition of PHA. Inhibition at points in the cell cycle other than G_0_/G_1 _are less likely to be seen since few cells progress to later stages of the cell cycle. In addition, it is possible that genetic mutations in PC-3 cells compromise some potential regulatory mechanisms. In particular, unlike lymphocytes, PC-3 cells lack functional p53 [28] which causes cell cycle arrest at G_1_/S by inducing p21^cip1^ that inhibits cyclin dependent kinases [29,30].

The mechanism by which OvUS inhibits cell cycle dynamics is not understood. One serpin has been identified which can affect cell cycle regulatory proteins. This serpin, myeloid and erythroid nuclear termination stage-specific protein (MENT), is a nuclear protein that inhibits cell proliferation through interactions with a nuclear protein with papain-like cysteine proteinase activity [31]. Inhibition of the proteinase prevents degradation of the cell cycle protein Rb although antiproliferative effects may depend more on other actions of MENT to mediate euchromatin condensation in an Rb-independent manner [31]. In any case, OvUS, is apparently without proteinase inhibitory activity and is an extracellular protein that is unlikely to achieve a nuclear localization. The antipepsin activity of uterine serpin is probably not biologically significant. OvUS is a very weak inhibitor of pepsin C (a 35 and 8 fold molar excess of OvUS was required to inhibit pepsin A and C [12]) C and the binding of OvUS to pepsin is electrostatic [15]. Moreover, pepsin shows an acidic pH optimum and is unlikely to be involved in cell proliferation under the conditions utilized.

A major point in the cell cycle regulated by OvUS is transition from G_0_/G_1 _to S phase: rOvUS decreased the proportion of cells in S phase in all experiments and increased the proportion of cells in G_0_/G_1 _at 24 h after treatment for PC-3 cells and at both times examined for lymphocytes. Ovine uterine serpin can bind to cell membranes [32] and, perhaps, OvUS inhibits proliferation by activation of signal transduction systems that inhibit transition from G_0_/G_1 _to S phase or prevents pro-proliferative molecules in culture medium from binding their receptors. Experiments with Rp-8-Cl-cAMPS, a selective inhibitor of cAMP-dependent type-I protein kinase A, indicated that effects of OvUS on proliferation of PHA-stimulated lymphocytes are not dependent on this kinase [24]. Studies to determine activation of other anti-proliferative signal transduction systems by OvUS are warranted.

## Conclusion

The present study indicates that the mechanism by which OvUS inhibits proliferation of PC-3 cells and lymphocytes involves cell cycle arrest and not, at least for PC-3 cells, apoptosis, cytotoxicity or inhibition of IL-8 secretion.

## Methods

### Materials

The human prostate cancer (PC-3) cell line was purchased from ATCC (Rockville, MD), the FreeStyle™ 293 expression medium, Dulbecco's Modified Eagle Medium Nutrient Mixture F-12 (DMEM-F12) and 0.25% Trypsin-EDTA were obtained from Gibco-Invitrogen (Carlsbad, CA), the RQ1 RNase-free DNase and the CellTiter-Glo^® ^Luminescent Cell Viability Assay kit were obtained from Promega, (Madison, WI), the DHL™ Cell Cytotoxicity Assay kit was from Anaspec (San Jose, CA) and the ELISA MAX™ Set Deluxe kit for human IL-8 was obtained from BioLegend (San Diego, CA). The *in situ *cell death detection kit [terminal deoxynucleotidyl transferase-mediated dUTP nick end labeling (TUNEL)] was purchased from Roche (Indianapolis, IN), the DNase-free RNase A was obtained from Qiagen (Valencia, CA), Precast Tris-HCl gradient Ready gels^® ^were from BioRad (Richmond, CA) and [^3^H]thymidine (6.7 Ci/mmol) was from ICN (Irvine, CA). The Prolong Antifade^® ^kit was purchased from Molecular Probes (Eugene, OR), Geneticin was from Research products international (Mount Prospect, IL), Centricon filter devices were from Millipore Corporation (Bedford, TX), niquel Sepharose chromatography medium (high performance) was from Amersham Biosciences (Piscataway, NJ), fetal bovine and horse serum from Atlanta Biologicals (Norcross, GA). Other reagents were obtained from either Fisher (Pittsburg, PA) or Sigma-Aldrich (St. Louis, MO).

### Purification of rOvUS

The His-tagged rOvUS was purified from conditioned medium of FreeStyle™ human embryonic kidney (HEK)-293F cells (Gibco-Invitrogen, Carlsbad, CA) transfected with a plasmid construct containing the gene for OvUS. Details of the cell line are provided elsewhere [25]. Cells were cultured continuously in selective medium [FreeStyle™ 293 expression medium containing 700 μg/ml of Geneticin^®^] at 37°C in a humidified 8% (v/v) CO_2 _incubator according to the manufacturer's recommendations. Conditioned medium containing rOvUS was diluted 1:1 (v/v) in binding buffer [20 mM sodium phosphate buffer, 35 mM imidazole, 0.3 M NaCl, pH 8.0] and loaded into a nickel Sepharose column that was pre-equilibrated with binding buffer. The His-tagged rOvUS was eluted with 20 mM phosphate buffer, 500 mM imidazole, 0.3 M NaCl, pH 8.0, concentrated and buffer-exchanged into Dulbecco's phosphate buffered saline (DPBS) using Centricon plus-20 concentration devices. Purity of the rOvUS was assessed by sodium dodecyl sulfate-polyacrylamide gel electrophoresis using precast 4–15% polyacrylamide Tris-HCl gradient gels. The protein concentration was determined by Bradford assay [33] using bovine serum albumin as standard.

For each experiment, rOvUS and the control protein, OVA, were added to culture wells of PC-3 cells or lymphocytes dissolved in DPBS. The vehicle control included addition of DPBS at the same volume as for rOvUS and OVA. The actual volume of protein or vehicle added varied between experiments but was generally 14–25 μl and never more than 50 μl. Cultures were set up so that the volume of DPBS was the same in all wells.

### PC-3 cell culture

The PC-3 cell line was cultured continuously in Dulbecco's Modified Eagle Medium Nutrient Mixture F-12 (DMEM-F12) supplemented with 10% (v/v) heat-inactivated fetal bovine serum, 200 U/ml penicillin and 2 mg/ml streptomycin at 37°C in a humidified 5% (v/v) CO_2 _incubator. For the IL-8 experiment only, the medium was modified to reduce the fetal bovine serum concentration to 4% (v/v). For all the experiments, cells were cultured in 75 cm^2 ^flasks until they reached 50–70% of confluence. Cells were then trypsinized, centrifuged at 110 × g for 5 min and resuspended in fresh complete medium. Cell viability was assessed by trypan blue exclusion and cell concentration was adjusted according to the requirements of each experiment.

### [^3^H]Thymidine incorporation by PC-3 cells

PC-3 cells (100 μl) were plated overnight at a final concentration of 1 × 10^5 ^cell/ml in a 96-well plate. Afterwards, various concentrations of rOvUS (0, 0.5, 1, 2, 4, 8, 16, 32, 64, 125 and 250 μg/ml) or vehicle were added to each well in a total volume (including additional culture medium) of 200 μl. After 48 h of culture, 0.1 μCi [^3^H]thymidine in 10 μl of culture medium were added. Cells were harvested 24 h after [^3^H]thymidine addition onto fiber-glass filters using a cell harvester (Brandel, Gaithersburg, MD). Filters were counted for radioactivity using scintillation spectrometry (Beckman Coulter Inc., Fullerton, CA). Each concentration of protein was tested in triplicate and the experiment was performed in six different replicates using a different batch of rOvUS for each replicate.

### Cell proliferation based on ATP content

Aliquots of 50 μl of PC-3 cells (1 × 10^5 ^cells/ml) were cultured for 24 h in a dark wall-clear bottom 96 well plate. Then, treatments consisting of vehicle (DPBS) or three different concentrations (50, 100 and 200 μg/ml) of rOvUS or a control protein (OVA) and culture medium added to bring the final volume to 100 μl. Additional control wells without cells were prepared to determine background. At 48 h after addition of treatments, ATP content per well was determined using the CellTiter-Glo^® ^Luminescent Cell Viability Assay kit according to the manufacturer's instructions. Briefly, 100 μl of the CellTiter-Glo^® ^reagent were added to each well, contents of the plate were mixed on a shaker for 2 min and then incubated at room temperature for 10 min. Chemiluminescence was quantified using a multi-detection microplate reader (FLX-800, BioTek, Winooski, VT). All treatments were performed in triplicates and the assay was performed on three different occasions using a different batch of rOvUS for each replicate.

### Cytotoxicity assay

The assay was based on the release of lactate dehydrogenase into culture medium following loss of cell membrane integrity accompanying cell death [34]. Procedures for cell culture and treatments were similar to those described for the ATP assay. At 48 h after addition of the treatments, release of lactate dehydrogenase into the medium was determined using the DHL™ Cell Cytotoxicity Assay kit following the vendor's instructions. Briefly, the plate was equilibrated at room temperature for 20 min before adding 10 μl of lysis solution or DPBS. To facilitate cell lysing, the plate was placed on a shaker for 2 min. A total of 50 μl lactate dehydrogenase assay solution was then added to each well. After 10 min at room temperature, the reaction was stopped using 20 μl of the stop solution and the fluorescence intensity was measured using a multi-detection microplate reader (FLX-800) with excitation and emission wavelengths of 530–560 nm and 590 nm, respectively. Percent cytotoxicity was calculated by dividing 100 × fluorescence from the unlysed cells by fluorescence of the lysed cells. For each assay, each treatment was performed in six wells. The assay was replicated five different times using a different batch of rOvUS for each replicate.

### TUNEL labeling

An aliquot of 100 μl of PC-3 cells were cultured overnight in chamber slides at a final concentration of 1 × 10^4 ^cells/ml. Then, treatments consisting of vehicle (DPBS), 50, 100 or 200 μg/ml rOvUS, or 200 μg/ml OVA were added and additional culture medium added to produce a final volume of 300 μl. After 24 and 48 h in culture with treatments, cells were washed with PBS/PVP [100 mM sodium phosphate pH 7.4, 0.9% (w/v) NaCl, 1 mg/ml polyvinyl pyrrolidone] and fixed with 4% (w/v) paraformaldehyde for 1 h at room temperature. Cells then were washed in PBS/PVP and stored at 4°C for the TUNEL (terminal deoxynucleotidyl transferase and fluorescein isothiocyanate-conjugated dUTP nick end labeling) procedure.

For TUNEL labeling, fixed cells were incubated for 1 h at room temperature with permeabilization solution [PBS, pH 7.4, 0.1 (v/v) Triton X-100, 0.1% (w/v) sodium citrate). After washing with PBS/PVP, slides were incubated with 50 μl of TUNEL reaction mixture containing terminal deoxynucleotidyl transferase and fluorescein isothiocyanate-conjugated dUTP, for 1 hour at 37°C. Positive controls were preincubated with RQ1 RNase-free DNase (50 U/ml) and negative controls were incubated without transferase. Slides were washed with PBS/PVP, incubated for 1 h with 50 μg/ml of RNase A and then for 30 min with propidium iodide (2.5 μg/ml) at room temperature. Slides were washed with PBS/PVP and Prolong Antifade^® ^was used to mount coverslips. Samples were observed using a Zeiss Axioplan 2 fluorescence microscope with dual filter (Carl Zeiss, Inc., Göttingen, Germany). Percent of cells with DNA fragmentation was determined by counting the total number of nuclei and total number of TUNEL-labeled nuclei at 10 different sites on the slide. The experiment was performed using three different batches of rOvUS.

### Secretion of IL-8

PC-3 cells (100 μl) were cultured in wells of a 96-well plate overnight at a final concentration of 1 × 10^5 ^cells/ml. Treatments were then added including vehicle (DPBS, similar volume as for rOvUS and OVA treatments), and three different concentrations of rOvUS and OVA (50, 100 and 200 μg/ml). The volume of each well was brought to 200 μl with culture medium. At 48 h after addition of treatments, cell culture supernatants were collected, centrifuged and stored at -20°C until ELISA for IL-8. Treatments were performed in triplicate for each assay; the experiment was repeated on three different occasions using three different batches of the recombinant protein. For the measurement of IL-8, the ELISA MAX™ Set Deluxe kit for human IL-8 was used according to the manufacturer's instructions using 100 μl of conditioned medium.

### Cell cycle analysis

PC-3 cells (100 μl) were cultured in 4 well plates at a final concentration of 4 × 10^5 ^cells/ml. After 24 h, treatments consisting of vehicle, 100 and 200 μg/ml rOvUS, and 200 μg/ml OVA were added with additional culture medium for a total volume of 400 μl. At 12 and 24 h after addition of treatments, cells were collected by trypsinization and washed with DPBS. Cells were fixed overnight in 70% (v/v) ethanol at 4°C, washed with DPBS and resuspended with 500 μl of staining solution [DPBS pH 7.4, 0.1% (v/v) Triton X-100, 0.05 mg/ml DNase-free RNase A, 50 μg/ml propidium iodide]. Cells were then analyzed by flow cytometry using a FACSort flow cytometer (Becton Dickinson, Franklin Lakes, NJ) and the red fluorescence of single events was recorded at wavelengths of 488 nm (excitation) and 600 nm (emission). Data were gated using pulse width and pulse area to exclude doublets, and the percent of cells present in each phase of the cell cycle was calculated using ModFITLT V3.1 software (Verity Software, Topsham, ME). The experiment was performed on three occasions with five different batches of rOvUS.

For the sheep lymphocyte experiment, mononuclear cells were purified by density gradient centrifugation from the buffy coat of heparinized peripheral blood collected by jugular venipuncture from non pregnant Rambouillet ewes [35]. After removing red blood cells by incubation with red cell lysis buffer (0.01 M Tris-HCl pH 7.5 containing 8.3 g/L of ammonium chloride), cell viability was assessed by trypan blue exclusion, and concentration adjusted to 4 × 10^6 ^cells/ml. Cells were then suspended in a culture medium consisting of Tissue Culture Medium-199 containing 5% (v/v) horse serum, 200 U/ml penicillin, 0.2 mg/ml streptomycin, 2 mM glutamine and 10^-5 ^M β-mercaptoethanol and aliquots of 100 μl cells cultured in 4 well plates in the presence or absence of 4 μg/ml PHA and with treatments of DPBS vehicle, 200 μg/ml rOvUS, and 200 μg/ml OVA. Total culture volume was 400 μl. After 72 and 96 h in culture at 37°C in a humidified 5% (v/v) CO_2 _incubator, lymphocytes were collected and washed with DPBS. Thereafter, lymphocytes were fixed and treated as described above. The experiment was performed separately for lymphocytes from four different sheep. Three different batches of rOvUS were tested for each sheep.

### Statistical analysis

Data were analyzed by least-squares means analysis of variance using the General Linear Models Procedures of SAS (SAS System for Windows, Version 9.0; SAS Institute, Cary, NC, USA). Error terms were determined based on calculation of expected mean squares with replicate considered random and other main effects considered fixed. For the cytotoxicity and IL-8 data, orthogonal polynomial contrasts were used to determine the linear and quadratic effects of rOvUS and OVA. In other analysis, the pdiff mean separation test of SAS was used to distinguish the difference of various levels of a treatment.

## Abbreviations

IL-8: Interleukin-8; Maspin: Mammary serine proteinase inhibitor; MENT: Myeloid and erythroid nuclear termination stage-specific protein; OVA: Ovalbumin; PC-3: Prostate cancer-3; PEDF: Pigment epithelium derived factor; PHA: Phytohemagglutinin; TUNEL: Terminal deoxynucleotidyl transferase (TdT) and fluorescein isothiocyanate-conjugated dUTP nick end labeling; US: Uterine serpin.

## Authors' contributions

MBP carried out all of the studies described, participated in experimental design and drafted the manuscript. PJH conceived of the study, participated in its design and coordination, and helped to draft the manuscript. Both authors read and approved the final manuscript.
